# Are Malnourished Inpatients Treated by Dietitians Active Participants in Their Nutrition Care? Findings of an Exploratory Study of Patient-Reported Measures across Nine Australian Hospitals

**DOI:** 10.3390/healthcare11081172

**Published:** 2023-04-19

**Authors:** Jack J. Bell, Alita Rushton, Kai Elmas, Merrilyn D. Banks, Rhiannon Barnes, Adrienne M. Young

**Affiliations:** 1 Allied Health, The Prince Charles Hospital, Brisbane, QLD 4032, Australia; 2 School of Human Movement and Nutrition Sciences, The University of Queensland, Brisbane, QLD 4072, Australia; 3 Dietetics and Food Services, Royal Brisbane and Women’s Hospital, Brisbane, QLD 4029, Australia; 4 Centre for Health Services Research, The University of Queensland, Brisbane, QLD 4072, Australia

**Keywords:** malnutrition, hospitals, dietitian, hospitals, nutrition therapy, participation, engagement

## Abstract

Background: Inpatient malnutrition is a key determinant of adverse patient and healthcare outcomes. The engagement of patients as active participants in nutrition care processes that support informed consent, care planning and shared decision making is recommended and has expected benefits. This study applied patient-reported measures to identify the proportion of malnourished inpatients seen by dietitians that reported engagement in key nutrition care processes. Methods: A subset analysis of a multisite malnutrition audit limited to patients with diagnosed malnutrition who had at least one dietitian chart entry and were able to respond to patient-reported measurement questions. Results: Data were available for 71 patients across nine Queensland hospitals. Patients were predominantly older adults (median 81 years, IQR 15) and female (n = 46) with mild/moderate (n = 50) versus severe (n = 17) or unspecified severity (n = 4) malnutrition. The median length of stay at the time of audit was 7 days (IQR 13). More than half of the patients included had two or more documented dietitian reviews. Nearly all patients (n = 68) received at least one form of nutrition support. A substantial number of patients reported not receiving a malnutrition diagnosis (n = 37), not being provided information about malnutrition (n = 30), or not having a plan for ongoing nutrition care or follow-up (n = 31). There were no clinically relevant trends between patient-reported measures and the number of dietitian reviews or severity of malnutrition. Conclusions: Malnourished inpatients seen by dietitians across multiple hospitals almost always receive nutritional support. Urgent attention is required to identify why these same patients do not routinely report receiving malnutrition diagnostic advice, receiving information about being at risk of malnutrition, and having a plan for ongoing nutrition care, regardless of how many times they are seen by dietitians.

## 1. Introduction

Malnutrition is a well-documented predictor of adverse patient-reported and healthcare outcomes and carries a heavy healthcare burden worldwide [1,2]. It is a broad term to describe a nutritional imbalance with undernutrition resulting from an intake deficit, increased requirements associated with a poor health state, complications from disease or illness, such as malabsorption, or a combination of these factors [3,4]. The determinants of malnutrition are diverse, multifactorial, and are often overlaid and interactive [4,5]. For example, a patient presenting with low intake, increased requirements, and reduced nutrient bioavailability, related to vomiting, malabsorption, inflammation, a restrictive diet, and a poor appetite, in the setting of a pancreatic tumor, with ongoing pain, repeated hospitalisations, and polypharmacy [4]. Interrelated determinants will be experienced differently depending on the context. For example, where this patient may live, along with their level of education, societal norms, and access to healthcare, will all impact on their nutritional outcomes [6]. Not surprisingly, malnutrition is considered a complex health problem that in many cases should not rely on any single nutrition care process or health professional to improve outcomes [7,8]. Where hospital inpatients are identified as malnourished or at risk of malnutrition, they should be thoroughly assessed, provided with a malnutrition diagnosis, offered nutritional interventions including nutrition support options, provided with information about malnutrition and treatment options, and offered the opportunity to engage in planning for ongoing nutritional care processes after they leave the hospital [9,10,11,12,13].

Despite increased attention to malnutrition screening and diagnosis, in many settings malnutrition is often underdiagnosed and undertreated [12,14]. The majority of patients with or at risk of malnutrition reside in the community or residential aged care settings. However, malnutrition is often first identified in those admitted to hospitals, where between 20 and 50% of admitted patients are identified as malnourished [1]. Where well-established malnutrition risk screening processes are in place, hospitals appear a logical point for malnutrition assessment and the commencement of intervention. However, improvements to screening and assessment approaches in hospitals are generating increasing demand. This is compounded by increasing acuity, a reduced length of stay, and a constrained human resource capacity to meet traditional service requirements [15,16].

Recent findings highlight that three in four inpatients with or at risk of malnutrition are not considered to require specialist care from a dietitian during their inpatient stay [17]. This raises the question whether inpatient dietitians are “best placed” to effectively treat all patients with or at risk of malnutrition, across all stages of the nutrition care process. Over the past decade, there has been increasing attention towards the need for a multidisciplinary, multimodal approach to the nutrition care of patients with or at risk of malnutrition [18,19,20,21]. The Systematised, Interdisciplinary Malnutrition Program for impLementation and Evaluation (SIMPLE) and Integrated Nutrition Pathway for Acute Care (INPAC) provide two examples of such approaches [16,17,22,23,24]. However, in practice, a systematised approach to malnutrition care is not widely adopted, and low-value care remains commonplace [14,16,17].

Clinician engagement of patients (or their carers) is a fundamental standard of care [25,26]. Malnutrition is no exception in this regard, and partnering with patients in planning, delivering, and evaluating their nutrition care is to be expected. This is underpinned by the transformational shift away from disease-centred care to a more personalised approach that should be driven by and evaluated using patient-reported measures, such as PREMs (Patient-reported Experience Measures) and PROMs (Patient-Reported Outcomes Measures) [27]. Patient-reported experience measures highlight gaps in inpatient food and mealtime care; however, there remains a lack of clarity regarding how well inpatients with or at risk of malnutrition are engaged in key nutrition care processes [28,29]. A recent study applying patient-reported measures demonstrated that a high proportion of “at risk” patients across diverse hospital settings did not report receiving a malnutrition diagnosis, the provision of information about malnutrition risk, or having a plan for ongoing follow-up [17]. However, these findings did not account for false positive nutrition screening nor adjust for patients that had not been seen by a dietitian.

This study therefore applied patient-reported measures to identify the proportion of malnourished patients with at least one documented dietitian consultation that reported receiving a malnutrition diagnosis, receiving information about malnutrition, and having a plan for ongoing nutrition care.

## 2. Materials and Methods

The Systematised Interdisciplinary Malnutrition Program for impLementation and Evaluation (SIMPLE) Phase II implementation program was a knowledge translation, quality assurance program aiming to scale and spread the initial SIMPLE program across hospitals in Queensland, Australia [16,17,30,31,32,33,34,35]. Led by an interdisciplinary knowledge translation team, the program engaged local dietetics and interdisciplinary ward/unit teams, the SIMPLE Champions Network and subgroups, and key site influencers to translate SIMPLE into new wards/units and hospitals.

This publication considers an exploratory subset analysis of a convenience sample of baseline audit data for SIMPLE Phase II hospitals [16,17]. Data were collected between January and July 2019 in 15 different wards across four metropolitan hospitals, four regional hospitals, and one rural hospital in Queensland, Australia [30]. Inclusion criteria for this subset analysis were limited to inpatients seen by a dietitian (as determined by at least one entry signed by an accredited practicing dietitian in the patient medical record), with a diagnosis of protein-energy malnutrition documented in the medical record by the treating dietitian for their current admission, who were able to respond to patient-reported measurement questions. Exclusion criteria were identified by the treating team as critically or terminally ill or less than 18 years of age.

All measures were collected within the scope of routine clinical practice improvement under ethics committee approval as a quality assurance project (HREC/18/QPCH/318). Malnutrition was diagnosed by accredited practicing dietitians applying validated tools in line with local procedures. Validated diagnostic tools applied across settings were the Subjective Global Assessment (SGA B—moderate or C—severe), ICD10-AM criteria (mild or moderate, severe), and/or Mini Nutritional Assessment-Short Form (MNA-SF score 7 or less—unspecified severity) [36,37,38]. The number of dietitian assessments was defined by the number of documented medical record entries signed by an accredited practicing dietitian. The length of stay was defined as the length of stay in days as of the day of the audit. Nutrition support was defined as the provision of at least one of the following: a high-protein and high-energy diet, high-protein and high-energy midmeals, oral nutrition supplements, or supplements as medicine, enteral, or parenteral nutrition, as documented in the medical record, medication chart, or food service system.

Following feedback from participating sites as part of the pre-implementation context analysis, patient-reported measures were adapted from the initial SIMPLE program (Table 1) [17]. Response options for these questions were yes, no, or unable to respond. Adaptations were iteratively made across a series of pragmatic action/reflection cycles prior to audit commencement. This process, facilitated by members of the SIMPLE Phase II Knowledge Translation team, engaged mapped stakeholders and teams [39,40,41]. Consensus for the final patient-reported measures was obtained from all sites and the SIMPLE Phase II Knowledge Translation Team prior to the commencement of the audit.

Trained dietitians, dietetics students under the supervision of a dietitian, and/or nutrition assistants (dietetics personnel) approached patients prior to discharge to apply a standardised peer audit tool after obtaining verbal consent. Sites were encouraged to use a “peer-reviewer” approach so that the audits were performed by dietetics personnel that were not the treating dietitian and to conduct audits on all patients in a ward, or if required, a random selection of patients. As a quality assurance activity, formal randomisation processes were not applied. A train the trainer approach utilised the SIMPLE champions network to support consistency of auditing. Supportive resources included a ward audit completion guide, data dictionary, and toolkit.

Table 1 highlights changes made to original SIMPLE patient-reported measures in response to site feedback.

Data were initially entered into Microsoft Excel by two members of the team and verified by a third person prior to statistical analysis undertaken using SPSS version 28. Continuous data were assessed for normality using the Shapiro–Wilk test. Due to the exploratory nature of the design and small numbers with no pre-determined power calculation, only descriptive statistical analyses were reported.

## 3. Results

**Figure 1:**
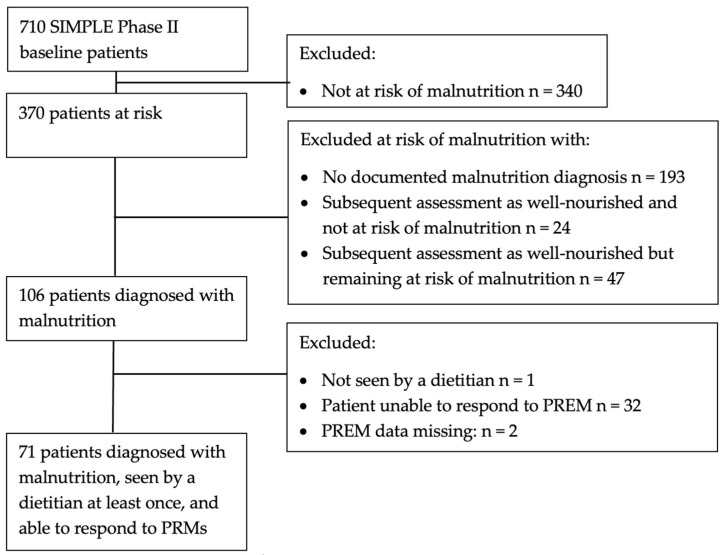
Participant flow diagram.

After excluding patients not at risk of malnutrition, those without a diagnosis of malnutrition, patients not seen by a dietitian at least once, and those unable to answer patient-reported measures, 71 patients had data available for this subset analysis (Figure 1).

The characteristics of included patients are described in Table 2. These highlight that malnourished patients already seen by dietitians at the time of the audit were likely to be older and female, with mild/moderate malnutrition, and were almost always receiving at least one form of nutritional support.

Patient-reported measures for key components of the nutrition care process are provided in Table 3. There were no clinically relevant trends between patient-reported measures and the median (IQR) number of dietitian reviews for risk identification discussion (No: 2 (2) versus Yes: 2 (2)), the provision of information (No: 2 (2) versus Yes: 2 (2)), or an ongoing plan for care or follow-up (No: 2 (2) versus Yes: 2 (2)).

Table 4 considers an exploratory combined measure that reported responses to all three patient-reported measures; that is, those who reported receiving a malnutrition diagnosis, receiving information about malnutrition, and having a plan for ongoing care or follow-up. Less than one in four malnourished patients seen by a dietitian reported a positive response to all three patient-reported measures. There were no clinically relevant trends between a positive response for all three patient-reported measures and the severity of malnutrition or the number of dietitian reviews. Although the small sample sizes and/or large interquartile ranges precluded the ability to draw conclusions for other measures, those of a younger age or who were male appeared more likely to respond positively to the combined measure.

## 4. Discussion

This study highlighted that almost all malnourished patients with at least 1 dietitian chart entry across 15 wards in 9 different hospitals in Queensland received oral nutrition support. However, it is perplexing as to why less than one in four of these patients reported receiving a malnutrition diagnosis, receiving information about being malnourished, and having a plan for ongoing nutrition care.

Malnutrition is coded internationally as a disease or related health problem with a profound deleterious influence on patients, healthcare systems, and the broader community [8,42,43]. Internationally recognised nutrition care recommendations and guidelines clearly articulate the need for inpatients with or at risk of malnutrition to (i) receive timely, systematic assessment and diagnostic advice; (ii) be involved in their care planning and decision making processes; (iii) receive multimodal and multidisciplinary interventions; (iv) receive nutritional information, education, or counselling; and (v) work with care providers to coordinate the transition of care into post-hospital settings [9,13,20,21]. The lack of engagement of patients across these core components of care raises potential ethical concerns regarding informed consent and shared decision making [11,14,44,45].

Are the results above due to an unreasonable and overwhelming demand for dietitians to deliver comprehensive, individualised nutrition care for all inpatients screened at risk of malnutrition prior to their discharge? There is acknowledgement of the increasing demands on dietitians working in hospitals, even prior to the COVID-19 pandemic, and that systematised and interdisciplinary approaches may provide part of the solution [9,13,17,20,21,46,47]. Given the complex nature of healthcare systems in an environment of scarcity, moving from knowledge to practice will be challenging [47]. However, the lack of any trends between patient-reported measures and the number of dietitian reviews suggests there are factors other than dietetic services overload.

Another alternate hypothesis is that the messaging is simply lost in translation. Effective clinical communication is a prerequisite for patient engagement. Patient engagement is shaped by the relationship between the patient and care provider and is a fundamental component of good clinical practice [48]. Results may indicate a need for improvements in dietitian communications with older patients, for example, around the nuancing of the medical terminology applied. They may also indicate inadequate dietitian supervision of students, for example, where gaps in dietetic student skills in engaging patients as part of client-centred care were not adequately addressed [49].

It is not uncommon to observe cognitive deficits in older, malnourished patients, particularly when placed in unfamiliar environments with high cognitive loads. The sampling approach applied in this study excluded those who were obviously unable to appropriately respond to the patient-reported measures. However, formal evaluations of patient cognition were not undertaken.

Another plausible explanation could simply be that older adults in hospitals routinely do not recall processes of engagement in their care. This is supported by the trends suggesting that those who reported receiving diagnostic information, receiving information about malnutrition, and having a plan for ongoing care were younger. However, if this is the case, a serious challenge is facing dietitians and the broader healthcare team. Are there more appropriate strategies to engage older patients in their nutrition care in such a way that they can recall such interactions? A balanced intervention would also need to ensure an equity of healthcare provision across ages [50].

A counter-intuitive finding remains open for interpretation. Why did the findings demonstrate males as more likely to respond positively to the combined patient-reported measures? This appears in contrast to many studies suggesting females are more likely to engage in their healthcare [51]. The study design precluded an opportunity to consider the gender balance of the dietetics workforce delivering care or those undertaking the audit. However, the well-recognised predominance of female dietetics personnel might also be considered a potential confounder to engagement between clinicians and patients [52,53].

The findings of this study support recent qualitative works highlighting that malnutrition remains a misunderstood diagnosis by both healthcare professionals and older adults across different settings [54]. There are many other potential reasons for this study’s findings than those highlighted above. Do patients who have already been commenced on nutrition support think they are no longer malnourished or no longer need to worry about it? Was the actual word “malnutrition” applied in the initial discussion between the dietitian (or student) or was a different term or phraseology applied, such as “you are a bit thin” or “you have lost some weight, haven’t you” [55]? Do patients only associate “malnutrition” with starved children in low- or middle-income countries or those in refugee or prisoner of war camps? Was the presence or exacerbation of other conditions leading to the cause for admission over-shadowing the co-diagnosis of malnutrition? Were patients, in an effort to maintain a positive affect, subconsciously denying the fact that circumstances of life now mean they are malnourished [56]?

All these and many more questions cannot be explained by this study and consequently require ongoing exploration. Given the retrospective exploratory study design, it would be improper to invoke firm conclusions regarding the underlying factors contributing to the results. However, what is clear is that patients remain in the dark about their malnutrition status, intervention options, and care plan. As noted elsewhere, effective communication matters when it comes to the complex technical term of malnutrition [57]. Dietetics personnel and the broader healthcare team must work together to engage patients, look across the malnutrition care journey and identify key touchpoints regarding who is best placed to communicate with patients about their nutrition care, and identify the most appropriate communication method [48,58].

As already noted, this study has considerable limitations, in particular the exploratory retrospective subset analysis. The results should be interpreted with some degree of caution. Whilst the initial patient-reported measures were developed by a team including a consumer with a lived experience of malnutrition, a more robust approach to the co-design of patient-reported measures could be another way to strengthen this work in the future. Similarly, the design did not allow for a consideration of the duration of time between dietitian consultation(s) and patient-reported measures, and so there may have been some influence on the accuracy of recall. The patient-reported measures were not specific to dietitians, and so whilst the findings demonstrate that dietitians are not communicating clearly about this, it seems clear than neither is the rest of the multidisciplinary team. Finally, the lack of a power calculation and small sample size precluded meaningful statistical analysis and so ongoing works are required to address this. The strengths lie in the multisite design and the ambitious approach to applying nutrition-focussed patient-reported measures in real-world settings.

## 5. Conclusions

The findings demonstrate that malnourished patients seen by dietitians across fifteen wards in nine different hospitals routinely receive oral nutrition support. However, regardless of the number of times dietitians see these patients, patient-reported measures do not reflect patient engagement across key components of the nutrition care process. Further exploratory works are required to unpack the implications of this for dietetics personnel and those they care for.

## Figures and Tables

**Table 1:** Patient-reported measures of key nutrition care processes.

	Original SIMPLE Patient-Reported Measures	Revised Patient-Reported Measures
PRM 1	Our nutrition screening process indicates you are at risk of malnutrition. Has anybody made you aware of this already?	I am here today because you have been identified as at risk of malnutrition. This is because you have a medical condition that places you at an increased risk of being malnourished, or it might be because you told a staff member that you have lost some weight, had a poor appetite, or you weren’t sure if you had lost any weight. Has anybody discussed this with you?
PRM 2	Have you seen or received any information or resources regarding nutrition?	Has someone provided you with any information about being at risk of malnutrition since you have been in hospital?
PRM 3	Has anybody discussed whether you need ongoing nutrition care and monitoring in hospital or after you leave hospital	Do you have a plan for ongoing nutrition care (prompts: dietitian or GP follow up)?

**Table 2:** Characteristics of included patients.

Characteristic		
Hospital location: n (%)		
Metropolitan	37	(52.1)
Regional or rural ^a^	34	(47.9)
Age in years: median (IQR) ^b^	81	(15)
Sex: n (%)		
Female	46	64.8
Male	25	35.2
Length of stay in days until time of audit: median (IQR)	6	(12)
Malnutrition severity: n (%)		
Mild/moderate or unspecified ^a^ severity	54	76.1
Severe	17	23.9
Number of dietitian chart entries: median (IQR)	2	(2)
Documented nutrition support: n (%)		
None or missing ^a^	3	4.2
One or more nutrition support processes	68	95.8

^a^ combined variables. ^b^ missing data (n = 14).

**Table 3:** Patient-reported measures for key components of the nutrition care process.

Patient-Reported Measures	No n (%)	Yes n (%)
You have been identified as at risk of malnutrition. Has anybody discussed this with you?	34 (47.9)	37 (52.1)
Have you been provided with any information about being at risk of malnutrition?	41 (57.7)	30 (42.3)
Do you have an ongoing plan for nutrition care or follow-up?	40 (56.3)	31 (43.7)

**Table 4:** Associations between patient characteristics and a combined measure of all patient-reported measures.

Patient Characteristics	Reported Receiving Malnutrition Diagnosis, Receiving Information about Malnutrition, and Having a Plan for Ongoing Care or Follow-Up	
	No (n = 56)	Yes (n = 15)
Hospital location: n (%)		
Metropolitan	31 (55.4)	6 (40.0)
Regional or rural	25 (44.6)	9 (60.0)
Age in years: median (IQR) ^a^	83 (13)	72 (26)
Sex: n (%)		
Female	40 (71.4)	6 (40.0)
Male	16 (28.6)	9 (60.0)
Length of stay in days until time of audit: median (IQR)	6 (11)	4 (16)
Malnutrition severity: n (%)		
Mild/moderate or unspecified	43 (76.8)	11 (73.3)
Severe	13 (23.2)	4 (26.7)
Number of dietitian chart entries: median (IQR)	2 (2)	2 (3)
Documented nutrition support: n (%)		
None or missing	2 (3.6)	1 (6.7)
One or more nutrition support processes	54 (98.2)	14 (93.3)

^a^ missing data (n = 14).

## Data Availability

As per the ethics exempted protocol, mixed methods data access remains restricted to members of the audit team.
